# Synthesis and Photocatalytic Performance of Five Organic–Inorganic Hybrid Supramolecules with Chain-like Organic Cations for Tetracycline Degradation

**DOI:** 10.3390/molecules30040817

**Published:** 2025-02-10

**Authors:** Changfu Chen, Xingxing Zhang, Chenfei Ren, Jiajun Yan, Kaijun Wu, Yi Yan, Xiru Song, Shufan Bu, Yunyin Niu

**Affiliations:** College of Chemistry and Pingyuan Laburatory, Zhengzhou University, Zhengzhou 450001, Chinaqw19837405013@stu.zzu.edu.cn (J.Y.); fanshubuchemistry@stu.zzu.edu.cn (S.B.)

**Keywords:** organic–inorganic hybrid supramolecules, photocatalytic degradation, water treatment, tetracycline pollution

## Abstract

In this paper, we synthesized a chain-like organic cation structure directing agent L·Cl_2_ by reacting triethylenediamine with 1,2-bis(2-chloroethoxy)ethane. We then used a room temperature volatilization method to react L·Cl_2_ with inorganic metal salts to synthesize five organic–inorganic hybrid supramolecules: {[L][HgI_4_]} (**1**), {[L][CdI_4_]} (**2**), {[(L)(Cu_2_I_3_)]·[CuI_2_]CH_3_CN}_n_ (**3**) {[L][CoCl_3_]_2_} (**4**), and {[L][Ce(NO_3_)_5_·(H_2_O)_2_]} (**5**) (L=1,1′-((ethane-1,2-diylbis(oxy))bis(ethane-2,1-diyl))bis(1,4-diazabicyclo [2.2.2]octan-1-ium) chloride). The compounds were characterized by X-ray diffraction, infrared spectroscopy, elemental analysis, and thermogravimetric analysis. Compounds **1**, **2**, and **5** are mononuclear anion compounds, while compound **3** is a 1D chain, and **4** is a binuclear anion compound. The results showed that 10 mg of compound **3** achieved 92.22% of tetracycline degradation efficiency in the initial TC solution pH = 7. The optimal conditions such as solution pH, catalyst dosage, and solution temperature for the photocatalytic degradation of TC wastewater by compound **3** were explored. Moreover, the photocatalytic degradation efficiency of compound **3** was above 86% each time after four cycles, indicating a good recyclability. The mechanism of photocatalytic degradation was also discussed.

## 1. Introduction

**Figure 1:**
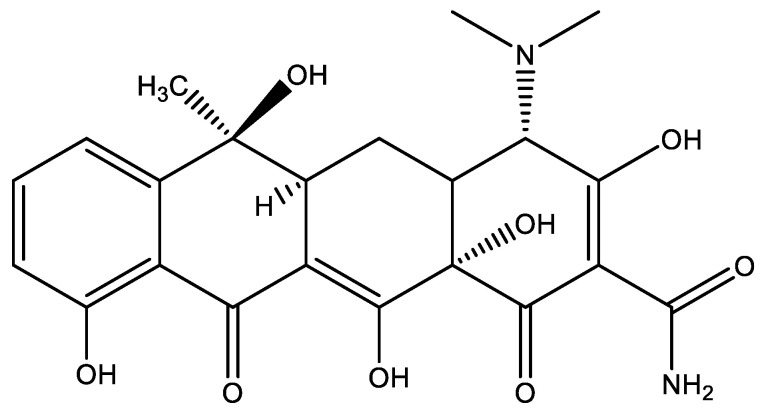
Chemical formula for TC.

Antibiotics play a unique role in disease prevention and control and growth promotion, which can promote the development of animal husbandry and protect human health. Tetracycline, as a typical antibiotic, can be used for the treatment of human and animal diseases, promoting the development of animal husbandry [1]. However, due to its high polarity, low volatility, and water solubility, tetracycline exhibits significant mobility and long-term persistence in the environment (Figure 1), making it difficult to degrade and leading to serious water pollution and environmental hazards [2,3]. Ye et al. reported that the concentration of tetracycline in pig farm wastewater ranged from 23.8–6857 μg/L [4]. The abuse of tetracycline can lead to strong allergic reactions or toxic side effects in the human body. It can also induce the generation of resistance genes and resistant microorganisms, which can then enter the human body through the food chain or other pathways, resulting in the decline of human immunity [5]. Tetracycline antibiotics present in feces and soil can migrate to plants through the “oil-water-plant root” medium transmission path, and their accumulation in organisms can partially inhibit the normal growth and development of plants [6,7]. Therefore, the efficient removal of tetracycline in water is of great practical significance. Through people’s long-term efforts and research, we have mastered the chemical, physical, and biological degradation methods [8]. Photocatalysis technology is considered a promising degradation technology due to its ecological and environmental friendliness [9,10,11,12,13].

Supramolecular chemistry covers the complex and ordered combination structure formed by molecules with specific functions through non-covalent weak interactions. It represents the sublimation of non-covalent molecular chemistry and is referred to as “chemistry beyond the molecule concept” [14,15,16]. It was initially proposed by the French scientist Jean-Marie Lehn, and is a multidisciplinary fringe science that has flourished in recent decades [17,18]. Supramolecular compounds play significant roles in many fields, such as catalysis, optics, and so on [19,20,21]. Supramolecular chemistry has provided important tools and methods for understanding and controlling molecular assembly and interactions. Moreover, the templating effect plays a crucial role in the synthesis of supramolecular compounds. In the construction of organic-inorganic hybrid supramolecular structures, organic cation templates not only balance anionic charges but also modify the structure. We have accumulated rich experience in this field of research [22,23,24,25]. The circular structure of 1,4-diazabicyclo[2.2.2]octane (DABCO) makes the orphan of two nitrogen atoms more naked and highly friendly, which allows 1,2-bis (2-chloroethoxy) ethane to react with it to form a cationic template. In addition, the nitrogen atoms of DABCO also easily coordinate with the metal, generating qualitatively stable molecular complexes. Based on this, DABCO and 1,2-bis(2-chloroethoxy)ethane were selected to synthesize a novel template cation, and reacted with inorganic metal salts to synthesize five new organic-inorganic hybrid compounds. The photocatalytic mechanism was investigated, and the degradation efficiency and kinetic properties were explored. After initial exploration, compound **3** is a potential photocatalytic catalyst.

## 2. Results and Discussion

### 2.1. Synthesis of Ligand and Compounds

#### 2.1.1. Synthesis of Ligand

**Scheme 1:**
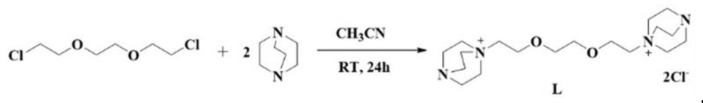
The syntheses of ligand L·Cl_2_.

In this paper, 1,4-diazabicyclo[2.2.2]octane and 1,2-di(2-chloroethoxy)ethane were selected to synthesize the organic cationic template L (Scheme 1).

#### 2.1.2. Synthesis of Compounds

**Scheme 2:**
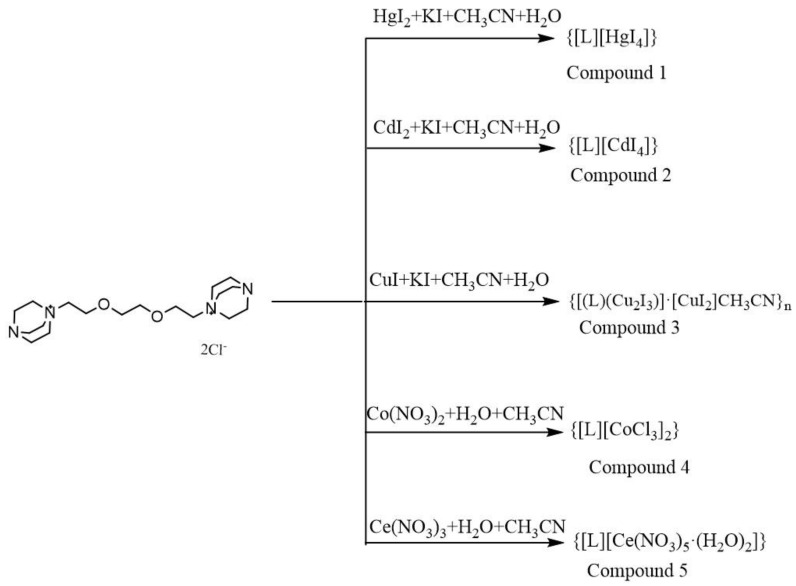
The syntheses of compounds **1**, **2**, **3**, **4**, and **5**.

Over the years, our laboratory has accumulated some experience in single crystals of synthetic complexes. In this paper, we used organic template L and metal salts to synthesize compounds **1**–**5** (Scheme 2) through solvent volatilization method.

### 2.2. Description of Crystal Structures

#### 2.2.1. Crystal Structure of {[L][HgI_4_]} (1)

**Figure 2:**
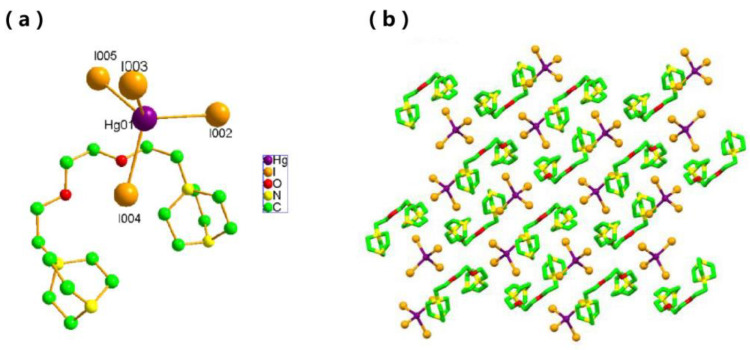
(**a**) Diagram of the smallest structural unit of compound **1**; (**b**) Crystal stacking diagram of compound **1** along axis b (Appendix B).

Single-crystal X-ray analysis shows that compound **1** belongs to the monoclinic system, with the *P*2_1_/n space group. Its asymmetric unit is composed by one [HgI_4_]^2−^ anion and one cationic ligand [L]^2+^, as depicted in Figure 2a. The metal ions Hg(II) are all coordinated in a distorted tetrahedral configuration, with bond lengths around the Hg(II) atoms as follows: Hg(01)-I(002) = 2.7633(7) Å, Hg(01)-I(003) = 2.7966(8) Å, Hg(01)-I(004) = 2.8083(8) Å, Hg(01)-I(005) = 2.7900(8) Å. The bond angles around Hg(II) are: I(002)-Hg(01)-I(003) = 110.46(2)°, I(002)-Hg(01)-I(004) = 106.21(2)°, I(002)-Hg(01)-I(005) = 115.72(3)°, (003)-Hg(01)-I(004) = 108.39(3)°, I(005)-Hg(01)-I(003) = 103.71(3)°, I(005)-Hg(01)-I(004) = 112.23(2)°, forming a slightly distorted tetrahedral coordination. The stacking diagram of compound **1** along the b-axis is illustrated in Figure 2b. Adjacent ligands combine irregularly to form a rectangular structure, which alternates with the inorganic anion part [HgI_4_]^2−^. The organic cations and inorganic anions are connected by weak electrostatic interactions, forming an infinite spatial stacking structure.

#### 2.2.2. Crystal Structure of {[L][CdI_4_]} (2)

**Figure 3:**
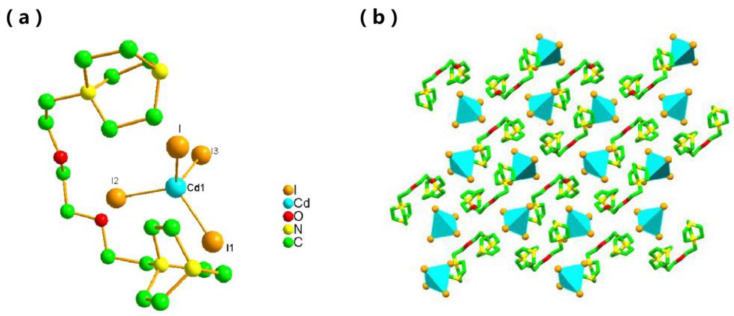
(**a**) Diagram of the smallest structural unit of compound **2**; (**b**) Crystal stacking diagram of compound **2** along axis b.

Single-crystal X-ray analysis shows that compound **2** belongs to the monoclinic crystal system, with the *P*2_1_/n space group. Its asymmetric unit is composed by one [CdI_4_]^2−^ anion and one cationic ligand [L]^2+^, as shown in Figure 3a. The inorganic anion portion, Cd^2+^, adopts a four-coordinate mode, with bond lengths to four iodine atoms measuring 2.767(8) Å, 2.780(8) Å, 2.791(8) Å, and 2.785(9) Å, and bond angles of 109.66(3)°, 106.63(3)°, 115.50(3)°, 108.34(3)°, 104.19(3)°, and 112.34(3)°, exhibiting a structure highly similar to compound **1**, forming a slightly distorted tetrahedral coordination. The stacking diagram of compound **2** along the a-axis is depicted in Figure 3b, where adjacent ligands combine irregularly to form rectangular structures, arranged in rows alternating with the inorganic anion portion [CdI_4_]^2−^. As with compound **1**, the organic cations and inorganic anions are connected by weak electrostatic interactions, forming an infinite spatial stacking structure.

#### 2.2.3. Crystal Structure of {[(L)(Cu_2_I_3_)]·[CuI_2_]CH_3_CN}_n_ (3)

**Figure 4:**
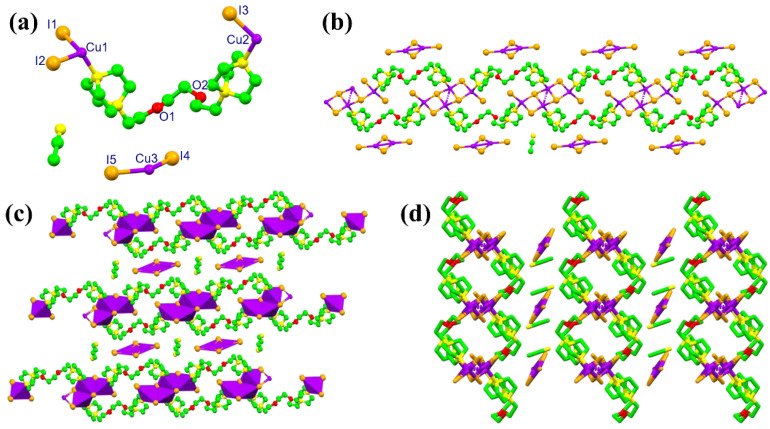
(**a**) Diagram of the smallest structural unit of compound **3**; (**b**) One-dimensional chain structure of compound **3**; (**c**) Crystal stacking diagram of compound **3** along axis a; (**d**) Crystal stacking diagram of compound **3** along axis b (H atom is omitted in the diagram).

Single-crystal X-ray analysis shows that compound **3** belongs to the triclinic crystal system, with the *P*-1 space group. As shown in Figure 4a, the minimal structural unit of compound **3** consists of a [(L)(Cu_2_I_3_)]^+^ cation, a free [CuI_2_]^−^ anion cluster, and a free acetonitrile solvent molecule. The N atoms of the ligands are connected to [CuI_2_]^−^ and [CuI] ions, with Cu_1_-I bond lengths of 2.677(6) and 2.614(5) Å in [CuI_2_]^−^, and a Cu_2_-I bond length of 2.577(6) Å in [CuI]. In the free [CuI_2_]^−^, Cu_3_-I bond lengths are 2.564(6) and 2.160(7) Å, with an I-Cu_3_-I bond angle of 113.60(4)°. As depicted in Figure 4b, the N atoms from two ligands are connected to the anion cluster [Cu_4_I_6_]^2−^, forming a rectangular pore-like structure with dimensions of 15.979 and 16.838 Å. Within the [Cu_4_I_6_]^2−^ cluster, Cu_1_ exhibits two coordination modes: in a five-coordinate mode, Cu_1_ interacts with three iodine atoms, one N atom from a ligand, and another Cu_1_^1^, forming an irregular tetrahedral configuration with a Cu_1_-Cu_1_^1^ bond length of 2.720 (10) Å; in a three-coordinate mode, Cu_1_^1^ interacts with two iodine atoms and Cu_1_, forming a triangular structure. Similarly, Cu_2_ within the [Cu_4_I_6_]^2−^ cluster also displays two coordination modes: a four-coordinate mode where Cu_2_ coordinates with three iodine atoms and one N atom from a ligand, forming a tetrahedral configuration; and a two-coordinate mode where Cu_2_^2^ coordinates with two iodine atoms, forming a tetrahedral edge-shared structure with Cu_1_. The [Cu_4_I_6_]^2−^ clusters form one-dimensional chain structures by connecting with ligands. The free [Cu_2_I_4_]^2−^ adopts a symmetric boat-shaped structure, formed by two [CuI_2_]^−^ units connected together, with a Cu_3_-Cu_3_^3^ bond length of 2.814 Å. The stacking diagram of compound **3** along the a-axis direction is illustrated in Figure 4c, where one-dimensional chains formed by [Cu_4_I_6_]^2−^ and ligands interact through π-π interactions and hydrogen bonding to generate a three-dimensional stacking structure, with free [Cu_2_I_4_]^2−^ and acetonitrile molecules intercalating between the chains. The stacking diagram along the b-axis direction, shown in Figure 4d, demonstrates a diamond-shaped structure formed by [Cu_4_I_6_]^2−^coordinating with two ligands, interconnected to form one-dimensional chain structures, while free [Cu_2_I_4_]^2−^ and acetonitrile molecules stack in a “Z” shape. These chain structures and the free “Z” structures intertwine to form a three-dimensional stacking structure.

#### 2.2.4. Crystal Structure of {[L][CoCl_3_]_2_} (4)

**Figure 5:**
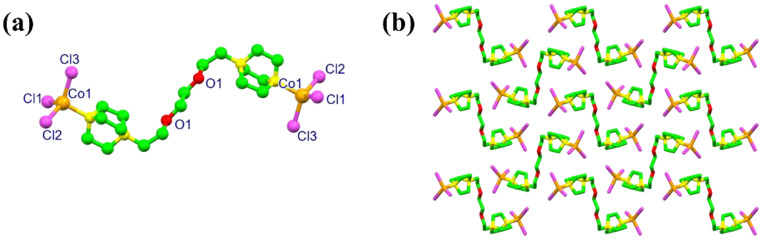
(**a**) Diagram of the smallest structural unit of compound **4**; (**b**) Crystal stacking diagram of compound **4** (H atom is omitted in the diagram).

Single-crystal X-ray analysis shows that compound **4** belongs to the monoclinic crystal system, as shown in Figure 5a, and consists of a cationic ligand [L]^2+^ coordinate to two anionic [CoCl_3_]^−^ to form a binuclear species. The metal ions Co(II) are all coordinated in a distorted tetrahedral configuration, with bond lengths around the Co(II) atoms as follows: Cl_1_-Co_1_ = 2.256(10) Å, Cl_2_-Co_1_ = 2.234(10) Å, Cl_3_-Co_1_ = 2.242(9) Å, and Co_1_-N_1_ = 2.088(16) Å. The bond angles around Co(II) are: Cl_1_-Co_1_-Cl_2_ = 131.1(3)°, Cl_2_-Co_1_-Cl_3_ = 115.91(4)°, Cl_1_-Co_1_-Cl_3_ = 112.43(4)°, N_1_-Co_1_-Cl_1_ = 108.11(5)°, N_1_-Co_1_-Cl_2_ = 104.22(5)°, and N_1_-Co_1_-Cl_3_ = 103.37(5)°. We can observe that the bond angles N_1_-Co_1_-Cl_2_ and N_1_-Co_1_-Cl_3_ are less than 109°28′, while the bond angle N_1_-Co_1_-Cl_1_ is very close to 109°28′. This discrepancy may be due to the steric hindrance caused by the ligands. As depicted in Figure 5b, compound **4** forms a three-dimensional stacking structure through intermolecular hydrogen bonding, presenting a wavy pattern.

#### 2.2.5. Crystal Structure of {[L][Ce(NO_3_)_5_^.^(H_2_O)_2_]} (5)

**Figure 6:**
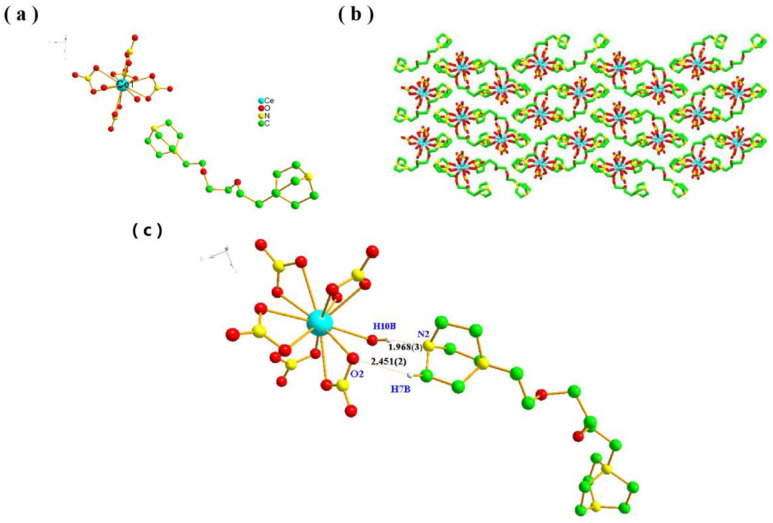
(**a**) Diagram of the smallest structural unit of compound **4**; (**b**) Crystal stacking diagram of compound **4** along axis b. (H atom is omitted in the diagram); (**c**) Hydrogen bond diagram for compound 5.

Single-crystal X-ray analysis shows that compound **5** belongs to the tetragonal crystal system, with the space group *P*4_1_2_1_2. As depicted in Figure 6a, the structural unit of compound **5** comprises a cationic ligand [L]^2+^ and a mononuclear [Ce(NO_3_)_5_∙(H_2_O)_2_]^2−^anions. Within the anionic [Ce(NO_3_)_5_∙(H_2_O)_2_]^2−^, the Ce(III) ions are coordinated to two oxygen atoms from each of the five nitrate groups and two oxygen atoms from water molecules. The bond lengths between Ce(III) ions and oxygen atoms from nitrate groups are 2.642(2), 2.702(2), 2.716(3), 2.656(2), and 2.627(3) Å, while the bond lengths to oxygen atoms from water molecules are 2.642(2) Å, indicating a symmetric structure for the anionic [Ce(NO_3_)_5_∙(H_2_O)_2_]^2−^. The stacking diagram of compound **5** along the b-axis is illustrated in Figure 6b, where two structural units overlap to form irregular diamond-shaped structures, and further aggregate into a three-dimensional structure through weak intermolecular interaction forces. In Figure 6c, weak O-H N and C-H O hydrogen bonding interactions exist between the organic cations and inorganic anions. The directional and selective nature of these hydrogen bonds allows the molecules to stack in an orderly direction during assembly, thereby forming a regular supramolecular structure. These hydrogen bonds also provide additional stability, making the supramolecular structure stronger.

### 2.3. Infrared Spectroscopy (IR) Analysis

**Figure 7:**
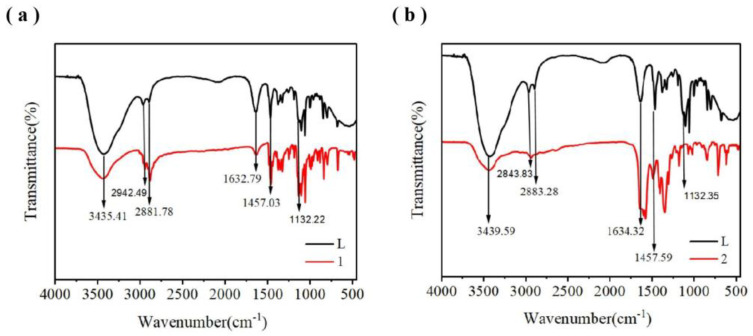

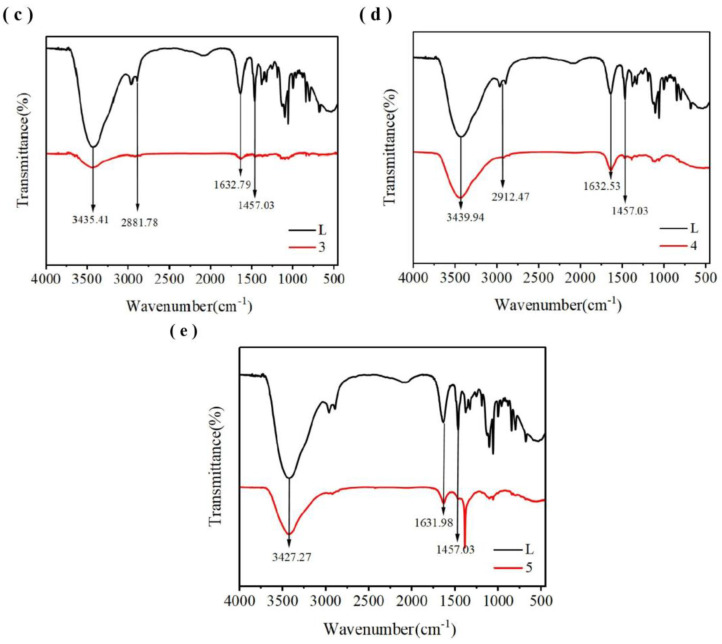
IR spectrogram of compounds **1**–**5**.

We characterized compounds **1**–**5** using infrared spectroscopy to analyze the characteristic functional group absorption peaks of the compounds. Taking compound **1** as an example, as shown in Figure 7, the ligand L synthesized experimentally was hygroscopic, as evidenced by the peak at 3435.41 cm^−1^ in the infrared spectrum. The peaks at 2942.49 cm^−1^ and 2881.78 cm^−1^ correspond to the stretching vibration peaks of -CH_2_- in the structure of ligand L. The absorption peak at 1632.79 cm^−1^ is attributed to the stretching vibration of C-N bonds, while the peak at 1457.03 cm^−1^ corresponds to the bending vibration of C-H bonds, and the peak at 1132.22 cm^−1^ is attributed to the stretching vibration of C-O bonds. These results are consistent with single crystal X-ray test results.

### 2.4. PXRD Analysis

**Figure 8:**
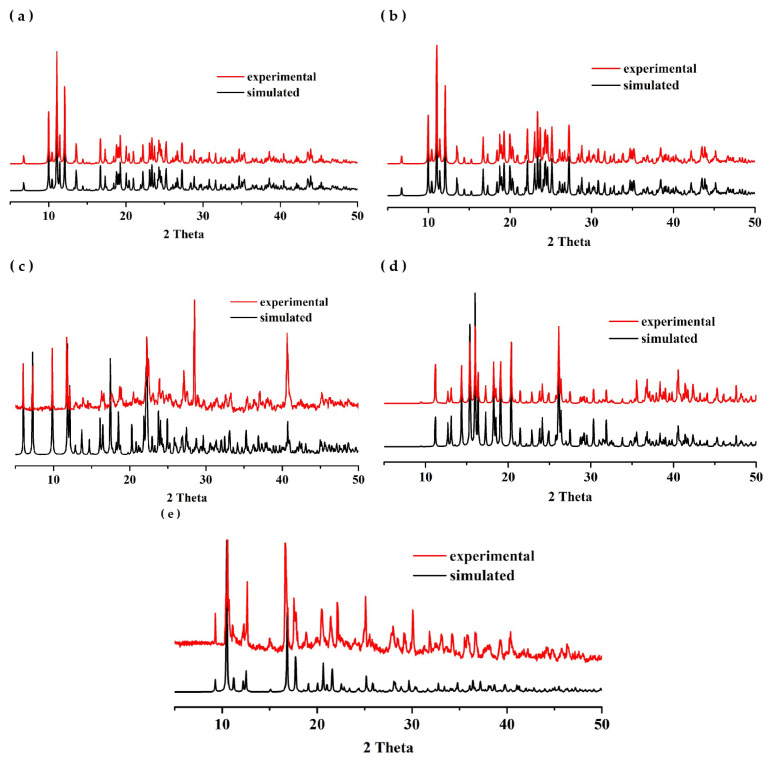
PXRD diagrams of compounds **1** (**a**), **2** (**b**), **3** (**c**), **4** (**d**), and **5** (**e**).

Based on the X-ray powder diffraction data shown in Figure 8, it can be observed that there is a high degree of fitting between the experimental and simulated data, indicating a high purity of compounds **1**–**5**.

### 2.5. Thermogravimetric (TG–DTG) Analysis

**Figure 9:**
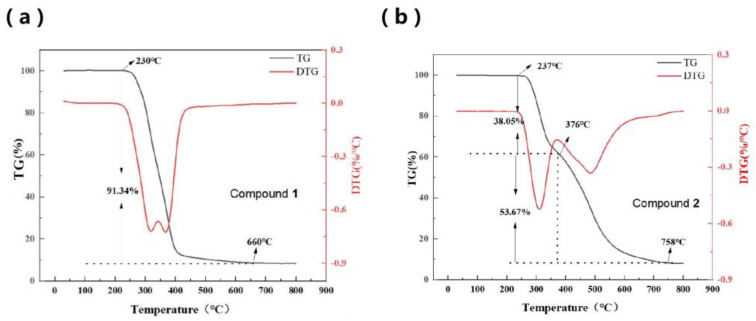

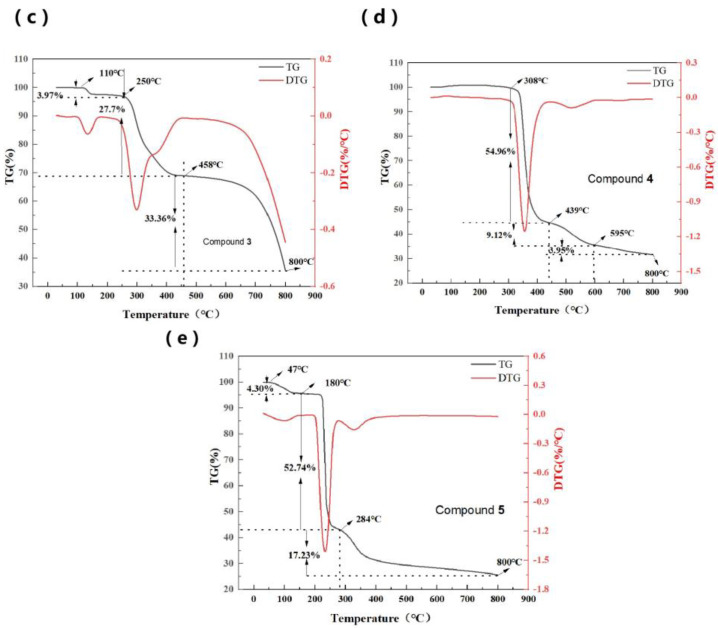
TG–DTA curve of compounds **1** (**a**), **2** (**b**), **3** (**c**), **4** (**d**), and **5** (**e**).

Under a controlled pure nitrogen atmosphere, the thermal stability behavior of compounds **1**–**5** was investigated within a temperature range of 30–800 °C, as depicted by their TG curves in Figure 9. It can be observed that compounds **1** and **2** mainly undergo mass loss in two stages. Prior to 230 °C, there is no significant change in mass, indicating the relative stability of the compounds’ structures. The first stage of mass loss primarily involves the decomposition of organic cations, while the second stage is mainly attributed to the thermal decomposition of inorganic metal salts. Compound **3** experiences mass loss in three stages. For compound **3**, within the temperature range of 100–250 °C, there is a first step weight loss attributed to the liberation of free CH_3_CN molecules, with an observed weight loss of 3.97%, close to the theoretical value of 3.90%.Mass loss between 250 and 430 °C is primarily attributed to the thermal decomposition of organic cation templates at high temperatures, while the continuous weight loss between 580 and 800 °C is ascribed to the complete collapse and decomposition of the anion framework. Compounds **4** and **5** exhibit mass loss primarily in a single stage, with no significant change in mass before 210 °C, indicating relatively stable structures. The mass loss is predominantly attributed to the complete collapse and volatilization of organic cation ligands and inorganic anion frameworks.

### 2.6. Band Gap Analysis

**Figure 10:**
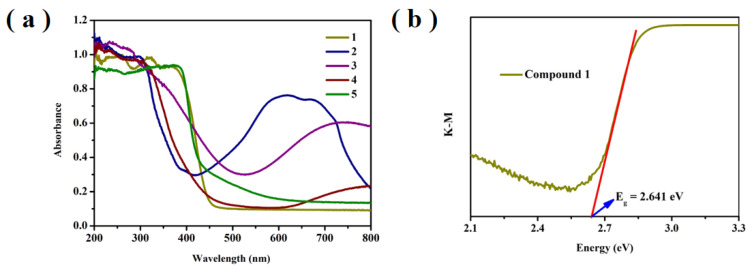

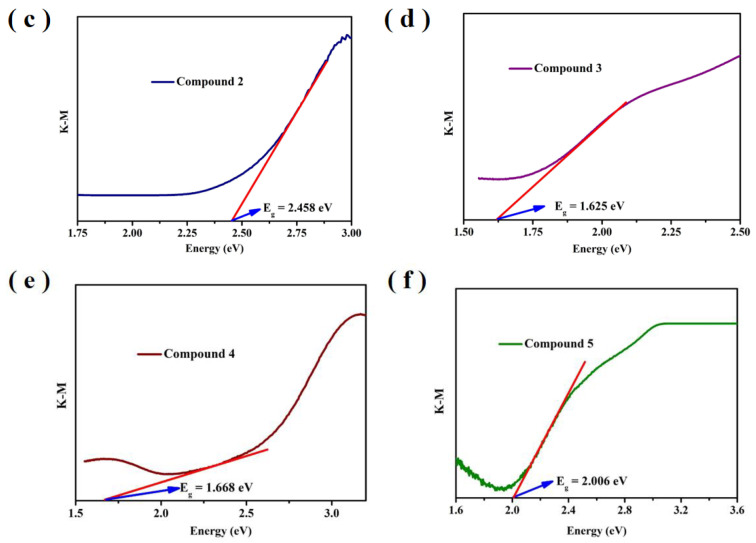
(**a**) Solid UV spectrum of compounds **1**–**5**. (**b**) band gap energy calculations of compound **1**. (**c**) band gap energy calculations of compound **2**. (**d**) band gap energy calculations of compound **3**. (**e**) band gap energy calculations of compound **4**. (**f**) band gap energy calculations of compound **5**.

The absorption region of the light was evaluated by the optical properties of the compounds, which were tested for compounds **1**–**5** using solid-state UV-visible diffuse reflection. As shown in Figure 10a, compounds **1**–**5** exhibit broad absorption in both the ultraviolet and visible regions, indicating their capability for light harvesting. This suggests that **1**–**5** can effectively capture light and generate more vitrification electrons and holes. The absorption coefficient α is calculated by using α = 4πk/λ from k (extinction coefficient). The variation in (*αhν*)^2^ with the function of incident photon energy (hν) is shown in Figure 10a–f. The relationship between band gap value and light absorption can be expressed by the following Formula (1)[26]. The band gap values *Eg* of **1-5** are 2.642, 2.458, 1.625, 1.668, and 2.006 eV, respectively.

(1) $$\alpha hv=A{\left(hv-Eg\right)}^{\frac{n}{2}}$$

where *A* is the proportionality constant, *Eg* is the band gap, *h* is Planck’s constant, *α* is the absorption coefficient, and *ν* is the optical frequency.

### 2.7. The Photocatalytic Properties of the Compounds **1**–**5**

**Figure 11:**
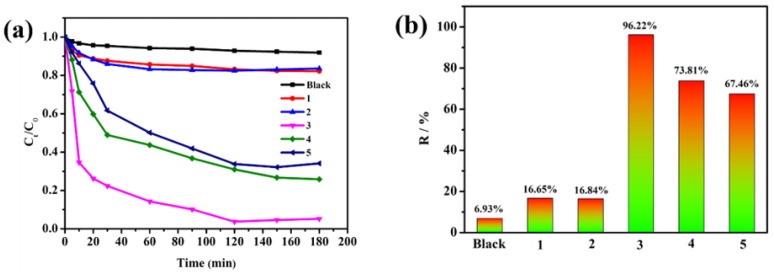
(**a**) The catalytic efficiency of catalysts **1**–**5** on TC. (**b**) Removal rate of TC.

With the increase in illumination time, the changes in the photocatalytic degradation of TC by compounds **1**–**5** were explored. Figure 11a,b depict the gradual decrease in TC concentration with increasing photocatalytic time. Compounds **1** and **2** exhibited less-than-ideal photocatalytic degradation of TC, reaching equilibrium at 60 min with degradation efficiencies of 16.65% and 16.84%, respectively. Compounds **4** and **5** achieved equilibrium in TC photocatalytic degradation at 150 min, with degradation efficiencies of 73.81% and 67.46%, respectively, indicating relatively higher degradation efficiencies for TC. Cao et al. combined SnS_2_ nanoparticles on the surface of the organic framework material of the zirconium, and the degradation rate of tetracycline (TC) reached 90% under visible light [27]. However, our synthesized compound **3** reached equilibrium in TC photocatalytic degradation at 120 min, with a degradation efficiency of 96.22%, demonstrating high photocatalytic efficiency. The gradual decrease in TC concentration with increasing photocatalytic time is attributed to the reduction in the amount of TC molecules binding to the active sites on the catalyst surface as illumination time increases, leading to a decrease in the contact probability between the active sites and TC, resulting in a slow increase in degradation efficiency. Through comprehensive analysis, compound **3** exhibited excellent photocatalytic degradation of TC. The higher efficiency of tetracycline degradation under the influence of compound **3** compared to other compounds may come from its small bandgap value(1.625eV). There is a close relationship between the bandgap of a semiconductor and its photocatalytic performance. The bandgap width determines the wavelength range of light that the semiconductor can absorb. The smaller the bandgap, the longer the wavelength of light that can be absorbed, which means that the semiconductor can absorb more visible light or even near-infrared light to make full use of sunlight. Further exploration of the optimal conditions and photocatalytic mechanism for TC degradation by compound **3** will be conducted next.

### 2.8. Degradation Kinetics of TC by Compound **3**

**Figure 12:**
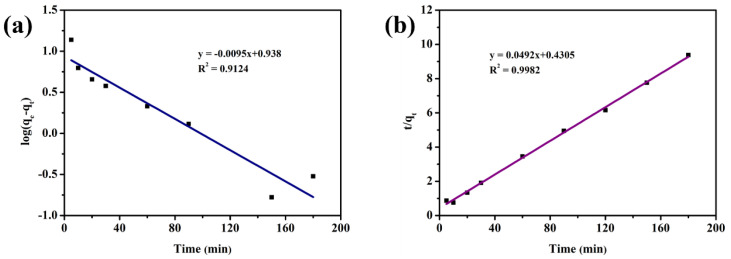
(**a**) A quasi-first-order kinetic model of the photocatalytic degradation of TC of compound 3. (**b**) quasi-second order kinetic model.

To further understand the intrinsic mechanism of compound **3** in the photocatalytic degradation of TC, the experimental results were fitted to pseudo-first-order and pseudo-second-order kinetic models to elucidate the actual degradation of TC. The pseudo-first-order and pseudo-second-order kinetic models for compound **3** are shown in Figure 12a,b, respectively. The regression coefficient R^2^ for the pseudo-first-order equation of compound **3** is 0.9124, and for the pseudo-second-order equation, it is 0.9982. Thus, it can be concluded that the photocatalytic degradation of TC by compound **3** is primarily governed by a pseudo-second-order kinetic model.

### 2.9. Effect of Other Factors on the Photocatalytic Degradation

#### 2.9.1. The Influence of Initial TC Solution pH

**Figure 13:**
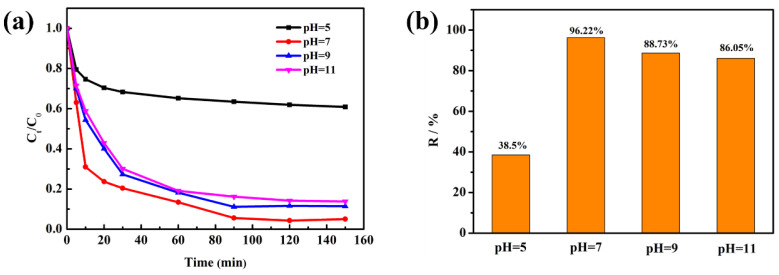
(**a**) Photocatalytic degradation of compound **3** under different pH conditions. (**b**) Removal rate of TC.

During the photocatalytic degradation of TC, the initial TC solution pH significantly affects the degradation process. Initially, the influence of pH on TC degradation efficiency was explored by taking 10 mg of compound **3** and 10 mL of a 20 mg·L^−1^ TC solution, and subjecting it to stirring conditions. A series of initial solution pH conditions, namely pH = 5, 7, 9, and 11, were set, and the degradation efficiency of TC was examined, as shown in Figure 13a,b. For compound **3**, the degradation efficiencies at pH = 5–11 were 38.5%, 96.22%, 88.73%, and 86.05%, respectively. It can be observed that with increasing pH, the efficiency of photocatalytic TC degradation initially increases and then decreases. Compound **3** exhibits the best degradation efficiency towards TC at pH 7. Different catalysts show varying photocatalytic degradation effects under different pH conditions, possibly due to differences in the charges carried by TC itself. Most TC exists in a positively charged state when the pH is below the Pka value of 3.30, primarily as TCH_3_^+^. At neutral pH, the main form is TCH_2_^0^, while at pH > 7.68, it becomes negatively charged, mainly existing as TCH^−^ or TC^2−^. The highest photocatalytic degradation efficiency of TC by compound **3** at pH 7 may be attributed to the electrostatic interaction between the charged compound **3** and TCH_2_^0^, leading to enhanced photocatalytic effects.

#### 2.9.2. The Effect of the Catalyst Dosage

**Figure 14:**
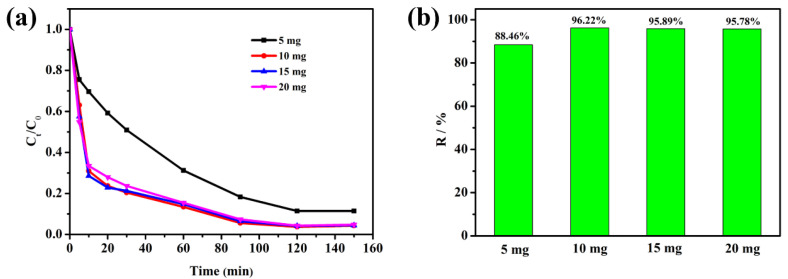
(**a**) Photocatalytic degradation of the TC by the amount of compound **3.** (**b**) Removal rate of TC.

The addition of different amounts of catalyst plays a crucial role in the photocatalytic degradation process. Under conditions of a TC solution concentration of 20 mg·L^−1^ and a volume of 10 mL, the degradation effects of various catalyst amounts were studied. The amounts of a series of catalysts were 5 mg, 10 mg, 15 mg, and 20 mg. The results of photocatalytic degradation are shown in Figure 14a,b. For catalyst **3**, as the amount of catalyst increased from 5 mg to 20 mg, the degradation efficiencies of TC were 88.46%, 96.22%, 95.89%, and 95.78%, respectively. Under conditions of a TC solution concentration of 20 mg·L^−1^ and a volume of 10 mL, the degradation effects of various catalyst amounts were studied. The amounts of a series of catalysts were 5 mg, 10 mg, 15 mg, and 20 mg. The results of photocatalytic degradation are shown in Figure 14a,b. For catalyst **3**, as the amount of catalyst increased from 5 mg to 20 mg, the degradation efficiencies of TC were 88.46%, 96.22%, 95.89%, and 95.78%, respectively. It is evident that the best catalytic effect is achieved when the amount of catalyst used is 10 mg. As the dosage of the catalyst continues to increase, there is no significant rise or fall in its catalytic action. However, when the catalyst dosage is reduced to 5 mg, a poorer catalytic effect can be observed, which may be due to the decreased absorption of photons at lower catalyst dosages, resulting in a slower degradation efficiency of TC.

#### 2.9.3. Temperature Effect

**Figure 15:**
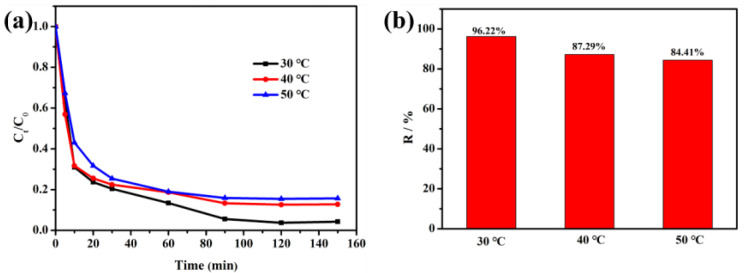
(**a**) TC photocatalytic degradation of compound **3** at different temperatures. (**b**) Removal rate of TC.

Temperature plays a crucial role in the photocatalytic degradation of TC. Under stirring conditions, the impact of different temperatures on the degradation efficiency of TC was explored in a 10 mg catalyst, 10 mL, 20 mg·L^−1^ TC solution. A series of temperatures including 30, 40, and 50 °C were investigated, and the results of photocatalytic degradation are shown in Figure 15a,b. For catalyst **3**, as the temperature increased from 30 °C to 50 °C, the degradation efficiencies of TC were 96.22%, 87.29%, and 84.41%, respectively. The catalytic effect showed a decreasing trend, with the highest catalytic effect observed at 30 °C. This indicates a decrease in the catalytic activity of catalyst 3 towards TC with increasing temperature.

#### 2.9.4. The Influence of the Coexisting Inorganic Anions

**Figure 16:**
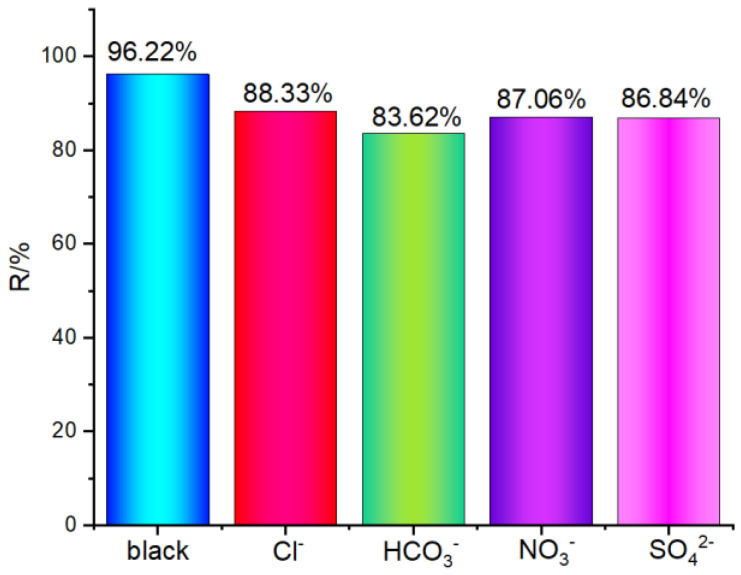
Diagram of the efficiency of TC removal of different 20 mg/L Cl^−^, 5 mg/L HCO_3_^−^, 5 mg/L NO_3_^−^, and 40 mg/L SO_4_^2−^ inorganic anions for compounds **3**.

The wastewater contains various salts, including a significant amount of sodium salts. This experiment investigated the effect of four coexisting anions, Cl^−^, HCO_3_^−^, NO_3_^−^, and SO_4_^2−^, on catalyst **3** on the removal of TC. The results, as shown in Figure 16, indicate that the addition of anions to the catalyst degradation system would inhibit the removal of TC. In the absence of added anions, the catalytic degradation of TC by catalyst **3** reached an efficiency of 96.22% after 120 minutes of photocatalysis. However, in the presence of Cl^−^, HCO_3_^−^, NO_3_^−^, and SO_4_^2−^, the degradation efficiencies of TC decreased to 88.33, 83.62, 87.06 and 86.84%, respectively. The reason for the decrease in TC degradation efficiency after the addition of certain anions may be attributed to the scavenging of ·OH by Cl^−^, HCO_3_^−^, NO_3_^−^, and SO_4_^2−^ anions, forming reactive species with lower activity than ·OH or ·O_2_^-^ ions (such as NO_3_·, SO_4_^−^, Cl·, PO_4_·^2−^, and HCO_3_·/CO_3_^−^).

### 2.10. Mechanism of the Photocatalytic Degradation

**Figure 17:**
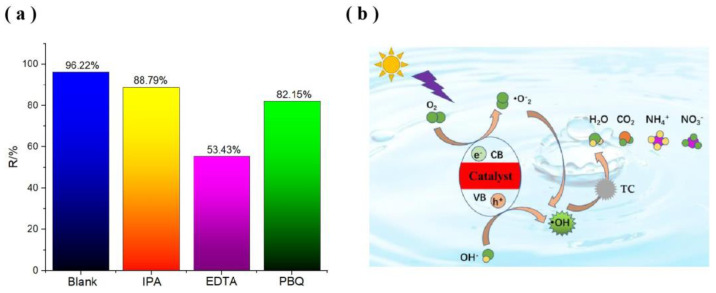
(**a**) Effect of different radical scavengers on TC removal of compounds 3. (**b**) photocatalytic mechanism of compounds.

In order to reveal and clarify the intrinsic mechanism of TC degradation, the roles of the primary active species were investigated. It is generally believed that the photo-induced primary active species include photogenerated holes (h^+^), superoxide anion radicals (O_2_^−^), and hydroxyl radicals (·OH), which can serve as catalysts in the photocatalytic reaction system. To further understand the role of the radicals generated by the catalyst in catalyzing the degradation of TC, the effect of adding different free radical scavengers on capturing TC in the photocatalytic reaction system was investigated under the same illumination conditions. The experiment employed three different quenching agents: isopropanol (IPA:1 mmol·L^−1^ ·OH scavenger), ethylenediaminetetraacetic acid disodium salt (EDTA-2Na:1 mmol·L^−1^, h^+^ scavenger), and 1,4-benzoquinone (PBQ: 1 mmol·L^−1^,·O_2_^−^ scavenger). The degradation efficiency of TC with the addition of different quenching agents is shown in Figure 17a. For catalyst **3**, after the addition of IPA, EDTA-2Na and PBQ, the TC degradation efficiency decreased from 96.22% to 88.79%, 53.43%, and 82.15%, respectively. The minimal variation of OH and O_2_^−^ during the photocatalysis indicates that their contribution to the degraded TC is weak. The significant reduction in TC photocatalytic degradation efficiency with h^+^ indicates that h^+^ is the primary active species involved in TC degradation. Through experimental exploration, the specific process of photocatalysis is hypothesized, as illustrated in Figure 17b. When the energy of the incident light equals or exceeds the semiconductor bandgap of the catalyst, under illumination conditions, electrons (e^−^) migrate from the valence band to the conduction band of the catalyst, generating electron-hole pairs (h^+^). O_2_ molecules combine with electrons to form ·O^2−^, which further transforms into hydroxyl radicals ·OH. Simultaneously, holes (h^+^) in the valence band combine with hydroxide ions, forming hydroxyl radicals ·OH. The hydroxyl radical ·OH exhibits strong oxidizing properties, capable of oxidizing TC molecules into other products such as H_2_O and CO_2_. The smaller the semiconductor band gap of the catalyst, the more conducive it is to electron transfer. Calculations of the band gap values for the compounds revealed that compound **3** has the smallest value, relative to others, at 1.625 eV, providing sufficient evidence for the relatively superior photocatalytic performance of compound **3**.

### 2.11. Cyclic and Regenerative Properties of Compounds

**Figure 18:**
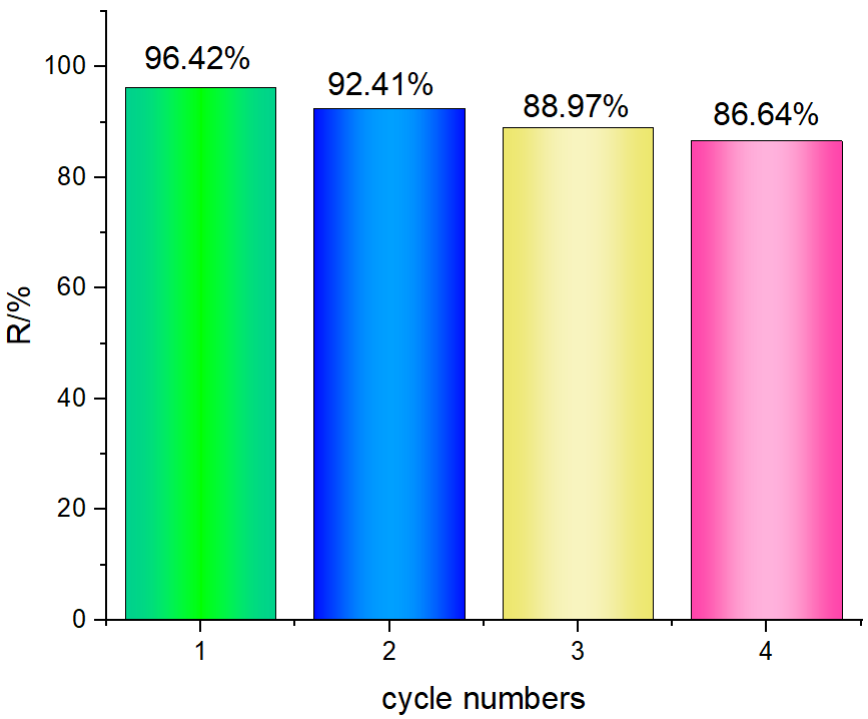
Four-cycle degradation efficiency plots of compound **3**.

The renewability and cyclability of the compounds are usually used to evaluate the use value of the catalyst. The catalyst was collected after each photocatalytic degradation, washed several times with deionized water to remove the residual TC, and then put back into the latest TC solution to start a new photocatalytic cycle experiment. Throughout the entire cycling process, all experimental conditions remain consistent. The cyclic results of photocatalytic degradation of TC by catalysts are shown in Figure 18. The decomposition efficiency of catalyst **3** for TC was not significantly reduced. After four cycles, the photocatalytic degradation efficiencies of catalyst **3** for TC were 96.22%, 92.41%, 88.97%, and 86.64%, respectively. The degradation results of the four cycles were above 86%. Therefore, compound **3** can become a potential catalyst for TC degradation.

## 3. Materials and Methods

### 3.1. Materials and Physical Measurements

All chemicals were of analytical grade and used without further purification.

Infrared (IR) spectroscopy: The instrument model is Bruker VECTOR 27, Karlsruhe, Germany. The infrared spectrum was measured in the range of 400–4000 cm^−1^ wave number by the KBr tablet method. Elemental Analysis (EA): The instrument model is Perkin-Elmer 240, Waltham, MA, USA. The content of C, H, and N elements in the compound was determined under room temperature conditions. Thermogravimetric (TG) Analysis: The instrument model is NETZSCHTG209, Selbu, Germany. The sample (5–10 mg) was heated from room temperature to 800 °C at a rate of 5 °C·min^−1^ under a nitrogen atmosphere with a gas flow rate of 30 cm^3^·min^−1^, and the mass change was recorded. X-ray Powder Diffraction (PXRD): The instrument model is X’Pert PRO, Nalytical, Netherlands. The light source is Cu-K α (λ = 1.5418 Å) at room temperature with a 2θ angular speed of 4 min^−1^, range 5–50°. X-Single crystal diffraction (SXRD): The instrument model is Bruker SMART CCD, Karlsruhe, Germany. After cultivating a single crystal, a crystal with uniform texture, suitable size and regular and bright shape was selected as the sample to be tested under the optical microscope. Liquid ultraviolet spectrum: The instrument model is UV-5500 PC, Primahh, Shanghai, China. Measurement of liquid absorption spectrum, quartz colorimetric dish, sweep range: 200–800 nm. Solid UV diffuse reflectance spectrum: The instrument model is UV-vis-NIR Cary 5000, Guangdong, China. The measurement was conducted at room temperature, with BaSO_4_ used as the blank reference, and the scanning range was from 200 to 800 nm.

### 3.2. Synthesis of Ligand

At room temperature, 1,2-di(2-chloroethoxy)ethane (0.5612 g, 3 mmol) was slowly added to 1,4-diazabicyclo[2.2.2]octane (1.0098 g, 9 mmol) dissolved in 30 mL acetonitrile. The mixed solution was reacted at RT for 24 h at room temperature to produce a white precipitate, washed three times with ethyl acetate and anhydrous ether, and dried under vacuum. Yield: 39.93% [28,29].

### 3.3. Synthesis of Compounds

#### 3.3.1. Synthesis of {[L][HgI_4_]} (1)

L·Cl_2_(0.0038 g, 0.01 mmol) in MeCN (4 mL) was added to HgI_2_(0.0045 g, 0.01 mmol) in MeCN (4 mL) in the presence of an appropriate amount of KI. The mixed solution was stirred for 10 min and then filtered. The filtrate was volatilized for 3 days at room temperature and the block-shaped buff transparent crystals were harvested. Yield: 49%. IR (KBr, cm^−1^): 3435.41 (m), 2942.49 (m), 2881.78 (m), 1632.79 (m), 1482.34 (m), 1457.03 (m), 1436.23 (m), 1398.21 (m), 1374.13 (m), 1349.54 (m), 1323.91 (m), 1246.70 (m), 1183.23 (m), 1146.88 (m), 1132.22 (m), 1114.49 (m), 1101.52 (m), 1072.94 (m), 1055.88 (s), 1000.07 (m), 988.26 (m), 972.75 (m), 958.49 (m), 921.12 (m), 908.48 (m), 880.34 (m), 834.50 (m), 793.51 (m), 670.51 (m), 551.23 (m), 534.61 (m), 487.48 (m), 477.07 (m), 423.31 (m). Elemental Anal. Calc. for C_18_H_36_N_4_O_2_HgI_4_ (1048.70): C, 20.57; H, 3.45; N, 5.38%. Found: C, 20.60; H, 3.43; N, 5.34%.

#### 3.3.2. Synthesis of {[L][CdI_4_]} (2)

The synthesis of Compound **2** is similar to that of Compound **1**, with CdI_2_ (0.0037 g, 0.01 mmol) replacing HgI_2_. The filtrate was volatilized for 6 days at room temperature, and the block colorless transparent crystals were harvested. Yield: 34%. IR (KBr, cm^−1^): 3439.59 (m), 2943.83 (m), 2883.28 (s), 1634.32 (m), 1482.97 (m), 1457.59 (s), 1437.03 (m), 1398.64 (m), 1374.45 (m), 1349.76 (m), 1324.29 (m), 1287.49 (m), 1247.02 (m), 1200.68 (m), 1183.37 (m), 1146.73 (s), 1132.35 (s), 1114.60 (s), 1101.41 (s), 1073.06 (m), 1055.89 (s), 1000.08 (m), 988.03 (m), 973.03 (m), 958.46 (m), 921.47 (m), 908.29 (m), 880.33 (m), 832.46 (s), 793.40 (m), 670.51 (m), 550.83 (m), 534.05 (m), 487.76 (m), 477.34 (m), 432.24 (m). Elemental Anal. Calc. for C_18_H_36_N_4_O_2_CdI_4_ (958.49): C, 22.53; H, 3.76; N, 5.84%. Found: C, 22.24; H, 3.75; N, 5.83%.

#### 3.3.3. Synthesis of {[(L)(Cu_2_I_3_)]·[CuI_2_]CH_3_CN}_n_ (3)

L·Cl_2_(0.0038 g, 0.01 mmol) in MeCN (4 mL) was added to CuI(0.0019 g, 0.01 mmol) in MeCN (4 mL) in the presence of an appropriate amount of KI, and then the mixed solution was stirred for 10 min and filtrated. The filtrate was volatilized for two weeks at 0 °C, and the clusters of colorless transparent crystals were harvested. Yield: 26%. IR (KBr, cm^−1^): 3879.81 (w), 3838.33 (w), 3796.02 (w), 3739.35 (w), 3714.38 (w), 3647.38 (w), 3445.62 (m), 2920.77 (w), 1747.48 (w), 1704.46 (w), 1688.39 (w), 1660.70 (w), 1635.04 (w), 1558.05 (w), 1456.50 (w), 1368.02 (w), 1313.69 (w), 1179.82 (w), 1142.34 (w), 1128.29 (w), 1099.56 (w), 1054.14 (w), 912.55 (w), 895.09 (w), 879.26 (w), 840.45 (w), 804.46 (w), 682.87 (w), 577.13 (w), 559.35 (w), 575.26 (w). Elemental Anal. Calc. For C_20_H_39_N_5_O_2_Cu_3_I_5_ (1206.68): C, 19.95; H, 3.25; N, 5.82%. Found: C, 19.90; H, 3.23; N, 5.80%.

#### 3.3.4. Synthesis of {[L][CoCl_3_]_2_} (4)

L·Cl_2_(0.0038g, 0.01mmol) in MeCN (4mL) was added to Co(NO_3_)_2_·6H_2_O (0.0029g, 0.01 mmol) in MeCN (4 mL) and two drops of water, and then the mixed solution was stirred for 10 min and filtrated. The filtrate was volatilized for 10 days at room temperature, and cylindrical blue transparent crystals were harvested. Yield: 4%. IR (KBr, cm^−1^): 3439.94 (m), 1632.53 (m), 1470.41 (w), 1384.12 (w), 1101.65 (m), 1058.53 (w), 524.93 (w). Elemental Anal. Calc. For C_18_H_36_N_4_O_2_Co_2_Cl_6_ (671.07): C, 32.19; H, 5.36; N, 8.34%. Found: C, 32.47; H, 5.11; N, 8.44%.

#### 3.3.5. Synthesis of {[L][Ce(NO_3_)_5_·(H_2_O)_2_]} (5)

The synthesis of Compound **5** is similar to that of Compound **4**, with Ce(NO_3_)_3_(0.0043 g, 0.01 mmol) replacing Co(NO_3_)_2_. The filtrate was volatilized for two weeks at room temperature and block-colorless transparent crystals were harvested. Yield: 33%. IR (KBr, cm^−1^): 3424.27 (m), 1631.98 (m), 1384.32 (m), 1057.94 (w), 546.20 (m). Elemental Anal. Calc. For C_18_H_40_CeN_9_O_19_ (826.71): C, 46.16; H, 5.29; N, 16.87%. Found: C, 46.18; H, 5.31; N, 16.93%.

### 3.4. X-Ray Crystallography Study

After cultivating a single crystal, a crystal with uniform texture, suitable size, and regular and bright shape was selected as the sample to be tested under the optical microscope. Compounds **1**–**5** were subjected for X-single crystal diffraction (SXRD) detection and data collection using Mo-Kα rays (λ = 0.71073 Å) or Cu-Kα rays (λ = 1.5418 Å), followed by absorption correction and resolved using SHELXTL-97, OLEX-2 and other program packages [30].The relevant crystallographic data are given in Table 1, and the main bond length and bond angle of compounds **1**–**5** are given in Table A1. The CCDC deposition numbers for compounds **1**, **2**, **3**, **4**, and **5** are 2288272, 2288273, 2288274, 2288277, and 2288275, respectively.

### 3.5. Catalytic Degradation of the Compounds **1**–**5**

To further evaluate the photocatalytic activity of compounds **1**–**5**, the typical antibiotic tetracycline was selected as the target contaminant for photocatalytic experiments under simulated visible light. A 300 W xenon lamp was used as the visible light source, and the solution pH was adjusted using 0.1 mol·L^−1^ HCl or NaOH solutions. The specific experimental procedure is as follows: First, 10 mg of catalyst was added to a TC solution (20 mg·L^−1^, 10 mL, pH = 7). At given time intervals, 3 mL of the degraded TC solution was collected, then centrifuged, and the supernatant was taken to measure the absorbance of the solution at 357 nm using a UV-visible spectrophotometer (UV-4100, Hitachi, Tokyo, Japan), further assessing the photocatalytic activity of compounds **1**–**5** towards TC. The calculation formula for the degradation efficiency (Formula (2)) is as follows [31].

(2) $$R\left(1-\frac{C_{t}}{C_{0}}\right)\times 100\%\;\;\;\;\;$$

where *C*_0_ is the initial concentrations (mg·L^−1^) of iodine; *C_t_* is the iodine concentrations (mg·L^−1^) at time t.

### 3.6. Kinetic Studies

In order to further understand the intrinsic mechanism of photocatalytic TC degradation by catalyst, the experimental results were fitted to the pseudo-first-order and pseudo-second-order kinetic models to determine the actual situation of TC degradation. The nonlinear forms of the quasi-first and quasi-second-order dynamical models are defined by Equations (3) and (4), respectively [32].

(3) $$\mathrm{lg}\left(q_{e}-q_{t}\right)=-\frac{K_{f}}{2.303}+lgq_{e}\;\;\;\;\;\;\;\;$$

(4) $$\frac{t}{q}=\frac{1}{q_{t}}t+\frac{1}{K_{s}q_{e}^{2}}\;\;\;\;\;\;\;\;\;\;\;$$

where *q_e_* is the equilibrium adsorption capacity (mg·g^−1^) of the adsorbent; *q_t_* is the adsorption capacity (mg·g^−1^) of the adsorbent at time t; *K_f_* is the pseudo-first-order model rate constants of adsorption (min^−1^); *K_s_* is the pseudo-second-order model rate constants of adsorption (g·mg^−1^·min).

## 4. Conclusions

In this paper, the organic cation ligand L was synthesized by reacting 1,2-bis(2-chloroethoxy)ethane with triethylenediamine. Five novel organic–inorganic compounds were self-assembled by reacting L with inorganic metal salts using a volatilization method. Through preliminary exploration, compound **3** exhibited the best photocatalytic degradation of TC. By further optimizing the experimental conditions of initial TC solution pH, catalyst amount, and temperature, we found that for catalyst **3**, in the initial TC solution, pH = 7.10 mg, catalyst was stirred at 30 °C for 120 min, and the degradation efficiency reached 92.22%. The thorough study of the photocatalytic degradation mechanism of TC by catalyst **3** revealed that h^+^ is the main active substance in the catalytic degradation of TC. After four cycles, catalyst **3** maintained a photocatalytic degradation efficiency of over 86% each time, demonstrating excellent recyclability and regenerability, making it a promising catalyst. In subsequent studies, we can increase the photocatalytic degradation experiment without a light source and explore the degradation performance of compound **3** for other antibiotic or dye contaminants.

## Figures and Tables

**Table 1:** Details of crystal data and structure refinement for compounds **1**–**5**.

Compounds	1	2	3	4	5
Empirical formula	C_18_H_36_N_4_O_2_HgI_4_	C_18_H_36_N_4_O_2_CdI_4_	C_20_H_39_N_5_O_2_Cu_3_I_5_	C_18_H_40_CeN_9_O_19_	C_18_H_40_CeN_9_O_19_
Formula weight	1048.70	958.49	1206.68	671.07	826.71
Crystal system	monoclinic	monoclinic	triclinic	monoclinic	tetragonal
Space group	P2_1_/n	P2_1_/n	P-1	*P2_1_/n*	*P4_1_2_1_2*
a/Å	15.8225(17)	15.868(4)	7.5099(2)	7.795(4)	9.9959(5)
b/Å	10.1284(11)	10.142(2)	15.0334(3)	15.796(6)	9.9959(5)
c/Å	18.088(2)	18.132(4)	15.2889(3)	11.581(5)	31.5263(16)
α/°	90	90	107.031(1)	90	90
β/°	101.909(2)	102.223(4)	91.8520(1)	95.647(16)	90
γ/°	90	90	94.4110(1)	90	90
Volume/Å^3^	2836.3(5)	2851.8(12)	1642.80(6)	1419.0(10)	3150.0(4)
Z	4	4	2	2	4
ρ/g.cm^3^	2.456	2.232	2.439	1.571	1.743
μ/mm^−1^	9.800	5.116	6.648	1.756	1.540
F(000)	1920.0	1784.0	1124	688	1684.0
Crystal size/mm^3^	0.2 × 0.15 × 0.1	0.2 × 0.15 × 0.1	0.26 × 0.24 × 0.17	0.36 × 0.18 × 0.1	0.25 × 0.22 × 0.17
T/K	296	296.15	298	299	296
Reflections collected	16779	16697	9984	34398	83555
Independent reflections	6388[Rint = 0.0582, Rsigma = 0.0764]			3261 [Rint = 0.0601, Rsigma = 0.0281]	3596[R_int_ = 0.0378, R_sigma_ = 0.0120]
Data/restraints/parameters	6388/0/262	6460/0/263	7594/0/325	261/0/145	3596/1/217
GOF on F^2^	0.998	1.039	1.032	1.038	1.093
Final R indexes [I ≥ 2σ (I)]	R_1_ = 0.0410, wR_2_ = 0.0794	R_1_ = 0.0506, wR_2_ = 0.1227	R_1_ = 0.0270, wR_2_ = 0.0518	R_1_ = 0.0295, wR_2_ = 0.0663	R_1_ = 0.0184, wR_2_ = 0.00471
Final R indexes [all data]	R_1_ = 0.0892, wR_2_ = 0.1069	R_1_ = 0.0737, wR_2_ = 0.1363	R_1_ = 0.0408, wR_2_ = 0.0553	R_1_ = 0.0510, wR_2_ = 0.0721	R_1_ = 0.0194, wR_2_ = 0.0475
Max/Min eÅ^-3^	0.85/−1.46	1.77/−1.90	1.013/−1.015	0.26/−0.21	0.33/−0.46

## Data Availability

All data generated or analyzed during this study are included in this article.

## Appendix A

**Table A1:** The main bond lengths and bond angles of compounds **1**–**5**.

Compound **1**					
Hg_1_-I_2_	2.7633(7)	Hg_1_-I_3_	Hg_1_-I_3_	Hg_1_-I_3_	Hg_1_-I_3_
Hg_1_-I_5_	2.7900(8)				
I_2_-Hg_1_-I_3_	110.46(2)	I_2_-Hg_1_-I_4_	106.21(2)	I_2_-Hg_1_-I_5_	115.72(3)
I_3_-Hg_1_-I_4_	108.39(3)	I_5_-Hg_1_-I_3_	103.71(3)	I_5_-Hg_1_-I_4_	112.23(2)
Compound **2**					
Cd_1_-I_1_	2.7670(8)	Cd1-I3	2.7800(8)	Cd_1_-I	2.7911(8)
Cd_1_-I_2_	2.7848(9)				
I_1_-Cd_1_-I_3_	109.66(3)	I_1_-Cd_1_-I_1_	106.63(3)	I_1_-Cd_1_-I_2_	115.50(3)
I_3_-Cd_1_-I	108.34(3)	I_3_-Cd_1_-I_2_	104.19(3)	I2-Cd_1_-I	112.34(3)
Compound **3**					
I_1_-Cu_1_	2.6765(6)	I_1_-Cu_1_	2.7073(6)	I_1_-Cu_2_	2.7940(6)
I_2_-Cu_1_	2.6140(5)	I_2_-Cu_2_	2.6828(6)	I_3_-Cu_2_	2.5771(6)
Cu_1_-Cu_1_	2.7197(10)	Cu_1_-N_2_	2.152(3)	Cu_2_-N_4_	2.157(3)
I_4_-Cu_3_	2.5758(6)	I_4_-Cu_3_	2.5636(6)	I_5_-Cu_3_	2.5160(7)
Cu_3_-Cu_3_	2.8143(11)				
Cu_1_-I_1_-Cu_1_	60.682(19)	Cu_1_-I_1_-Cu_2_	111.704(17)	Cu_1_-I_1_-Cu_2_	75.205(16)
Cu_1_-I_2_-Cu_2_	78.152(18)	I_1_-Cu_1_-I_1_	119.318(19)	I_1_-Cu_1_-Cu_1_	59.10(2)
I_1_-Cu_1_-Cu_1_	60.218(19)	I_2_-Cu_1_-I_1_	105.765(19)	I_2_-Cu_1_-I_1_	108.968(19)
I_2_-Cu_1_-Cu_1_	126.22(3)	N_1_-Cu_1_-I_1_	103.26(8)	N_1_-Cu_1_-I_1_	104.41(8)
N_1_-Cu_1_-I_2_	115.53(8)	N_1_-Cu_1_-Cu_1_	118.24(8)	I_2_-Cu_2_-I_1_	100.745(19)
I_3_-Cu_2_-I_1_	119.20(2)	I_3_-Cu_2_-I_2_	115.44(2)	N_4_-Cu_2_-I_1_	102.70(8)
N_4_-Cu_2_-I_2_	107.46(8)	N_4_-Cu_2_-I_3_	109.96(8)	C_1_-N_1_-Cu_1_	113.8(2)
C_3_-N_1_-Cu_1_	110.9(2)	C_5_-N_1_-Cu_1_	109.5(2)	C_14_-N_4_-Cu_2_	111.6(2)
C_16_-N_4_-Cu_2_	113.4(2)	C_18_-N_4_-Cu_2_	108.5(2)	Cu_3_-I_4_-Cu_3_	66.40(2)
I_4_-Cu_3_-I_4_	113.60(2)	I_4_-Cu_3_-Cu_3_	57.01(2)	I_4_-Cu_3_-Cu_3_	56.59(2)
I_5_-Cu_3_-I_4_	120.52(2)	I_5_-Cu_3_-I_4_	125.29(2)	I_4_-Cu_3_-Cu_3_	172.32(4)
I_1_-Cu_1_	2.6765(6)	I_1_-Cu_1_	2.7073(6)	I_1_-Cu_2_	2.7940(6)
I_2_-Cu_1_	2.6140(5)	I_2_-Cu_2_	2.6828(6)	I_3_-Cu_2_	2.5771(6)
Compound **4**					
Co_1_-Cl_11_	2.2557(10)	Co_1_-Cl_12_	2.2341(10)	Co1-Cl_13_	2.2423(9)
Co_1_-N_1_	2.0879(16)				
Cl_2_-Co_1_-Cl_1_	111.79(4)	Cl_2_-Co_1_-Cl_3_	115.91(4)	Cl_3_-Co_1_-Cl_1_	112.43(4)
N_1_-Co_1_-Cl_1_	108.11(5)	N_1_-Co_1_-Cl_2_	104.22(5)	N_1_-Co_1_-Cl_3_	103.37(5)
Compound **5**					
Ce_1_-O_2_	2.642(2)	Ce_1_-O_2_^1^	2.642(2)	Ce_1_-O_3_^1^	2.702(2)
Ce_1_-O_3_	2.702(2)	Ce_1_-O_5_	2.716(3)	Ce_1_-O_5_^1^	2.716(3)
Ce_1_-O_7_	2.656(2)	Ce_1_-O_7_^1^	2.656(2)	Ce_1_-O_8_	2.627(3)
Ce_1_-O_8_^1^	2.627(3)	Ce_1_-O_10_	2.564(2)	Ce_1_-O_10_^1^	2.564(2)
O_2_-Ce_1_-O_2_^1^	164.31(11)	O_2_^1^-Ce_1_-O_3_	130.11(8)	O2^1^-Ce_1_-O_3_^1^	47.33(8)
O_2_-Ce_1_-O_3_	47.33(8)	O_2_-Ce_1_-O_3_^1^	130.11(8)	O2^1^-Ce_1_-O_5_	103.19(9)
O_2_^1^-Ce_1_-O_5_^1^	61.28(9)	O_2_-Ce_1_-O_5_^1^	103.19(9)	O_2_-Ce_1_-O_5_	61.28(9)
O_2_-Ce_1_-O_7_	66.93(7)	O_2_-Ce_1_-O_7_^1^	106.90(8)	O_2_^1^-Ce_1_-O_7_	106.90(8)
O_2_^1^-Ce_1_-O_7_^1^	66.93(7)	O_3_-Ce_1_-O_3_^1^	165.89(12)	O_3_-Ce_1_-O_5_^1^	102.95(9)
Ce_1_-O_2_	2.642(2)	Ce_1_-O_2_^1^	2.642(2)	Ce_1_-O_3_^1^	2.702(2)

## Appendix B

The complete crystallography data of compounds **1**–**5** can be obtained free of charge from The Cambridge Crystallographic Data via www.ccdc.cam.ac.uk/structures (accessed on 4 January 2025), with CCDC numbers of 2288272 (for compound **1**), 2288273 (for compound **2**), 2288274 (for compound **3**), 2288277 (for compound **4**), and 2288275 (for compound **5**), respectively.
